# Synergic pro-apoptotic effects of Ferulic Acid and nanostructured lipid carrier in glioblastoma cells assessed through molecular and Delayed Luminescence studies

**DOI:** 10.1038/s41598-020-61670-3

**Published:** 2020-03-13

**Authors:** Rosaria Grasso, Paola Dell’Albani, Claudia Carbone, Michela Spatuzza, Roberta Bonfanti, Giovanni Sposito, Giovanni Puglisi, Francesco Musumeci, Agata Scordino, Agata Campisi

**Affiliations:** 1 Department of Physics and Astronomy “Ettore Majorana”, University of Catania, 95123 Catania, Italy; 2 Istituto Nazionale di Fisica Nucleare, Laboratori Nazionali del Sud, 95123 Catania, Italy; 3 Institute for Biomedical Research and Innovation, Italian National Research Council, 95126 Catania, Italy; 4 Department of Drug Sciences, Laboratory of Drug Delivery Technology, University of Catania, 95123 Catania, Italy; 5 Oasi Institute for Research on Mental Retardation and Brain Aging (IRCCS), 94018 Troina, Italy; 6 Department of Drug Sciences, Section of Biochemistry, University of Catania, 95123 Catania, Italy

**Keywords:** Cancer therapy, CNS cancer, Biological physics, Drug delivery

## Abstract

Herein, we assessed the effect of Ferulic Acid (FA), a natural antioxidant with anti-cancer effect, on the human glioblastoma cells through molecular and Delayed Luminescence (DL) studies. DL, a phenomenon of ultra-week emission of optical photons, was used to monitor mitochondrial assessment. The effect of FA loaded in nanostructured lipid carriers (NLCs) was also assessed. To validate NLCs as a drug delivery system for glioblastoma treatment, particular attention was focused on their effect. We found that free FA induced a significant decrease in c-Myc and Bcl-2 expression levels accompanied by the apoptotic pathway activation. Blank NLCs, even if they did not induce cytotoxicity and caspase-3 cleavage, decreased Bcl-2, ERK1/2, c-Myc expression levels activating PARP-1 cleavage. The changes in DL intensity and kinetics highlighted a possible effect of nanoparticle matrix on mitochondria, through the involvement of the NADH pool and ROS production that, in turn, activates ERK1/2 pathways. All the effects on protein expression levels and on the activation of apoptotic pathway appeared more evident when the cells were exposed to FA loaded in NLCs. We demonstrated that the observed effects are due to a synergic pro-apoptotic influence exerted by FA, whose bio-availability increases in the glioblastoma cells, and NLCs formulation.

## Introduction

Glioblastoma multiforme (GBM), also known as grade IV astrocytoma, represents the most prevalent and aggressive brain cancer. It is characterized by glial cells and has finger-like tentacles that infiltrate the brain, which make them very difficult to remove with surgical procedures. GBM exhibits a high level of resistance to conventional chemotherapy and radiotherapy, also due to the existence of blood-brain barrier (BBB), glioma stem cells and complex network of multiple modified signalling pathways^1^. The most frequent aberrant expression is represented by the dysregulation of extracellular signal-regulated protein kinase (ERK), which is associated with poor survival of the patients. The ERK isoforms (p42/44 or ERK1/ERK2) by interacting with specific phosphorylation substrates, play a pivotal role in the control of several cellular processes involved in proliferation, as well as activation of transcription factors, apoptosis and the control of cellular process^2,3^. In addition, the transcription factor c-Myc has been recognized as an important regulator of stem cell biology implicated with GBM malignancy and stemness^4^, as it contributes to proliferation, growth and survival of GBM stem cells^5^. GBM has been also related to the impairment of mitochondrial metabolic capacity, which leads to the alteration in energy production^6,7^ and is characterized by an overexpression of Bcl-2^8^. This protein can regulate transition pores permeability of the outer mitochondrial membrane and block pro-apoptotic proteins^9^. Furthermore, it has been identified a novel interaction between Bcl-2 and (ADP-ribose) polymerase (PARP)^10^, the best-known protein which plays role in the repair of DNA single-strand breaks and DNA base excision. In particular, PARP-1 is highly expressed in several types of cancers, including glioma. In patient affected by GBM, it is localized into the nucleus^11,12^, and supports the function played by the protein in the maintenance of genome integrity and in resistance to apoptosis^11^.

However, the mitochondria role in the chemo-resistance development in GBM is not well clarified^13^. An innovative technique that could improve the mitochondria assessment is Delayed Luminescence (DL), a photo-induced, prolonged in time, phenomenon of ultraweak emission of optical photons. Starting from the first observation in the 50s of this phenomenon in plant by Strehler and Arnold^14^, a wide Literature on the application aspects of DL, also sometime referred as Delayed Fluorescence or Delayed Light Emission, was produced connecting it to the activity of Photosystem II in cyanobacteria and plants^15–17^. Starting in the early 2000s DL from mammalian cells has been also measured showing potential utility for a wide variety of applications. Actually it has been shown that DL can discriminate, in a quick and non-invasive way, between normal and tumour cells^18–20^. Furthermore, it has been shown the possibility of monitoring cell status, cell cycle progression and evaluating the pro-apoptotic capacity of certain treatments^21–23^ along with the possibility of measuring mitochondrial oxygen tension^24,25^. DL has been related to the electron transfer steps in Mitochondrial Respiratory Chain (MRC) Complex I^26^, so assessing its potentialities in studying mitochondrial functionality. Worth to note that MRC Complex I represents the counterpart of Photosystem II for electron transfer. From a theoretical point of view DL has been associated to the existence of triplet- or metastable-state species, whose lifetimes are intrinsically long, along with the formation of coherent collective electron and exciton states, in general, and solitons (electrosolitons) in particular^27,28^.

Several findings have focused on the use as anticancer agents of natural antioxidant compounds, that in a direct^29,30^ or indirect^31^ way might have mitochondria as target^7^. In previous studies, we found that Ferulic Acid (4-hydroxy-3-methoxycinnamic acid, FA), a natural antioxidant isolated from *Ferula foetida* L.^32,33^, was able to activate the apoptotic pathway in U-87 MG cell line cultures^34^. Furthermore, we observed that this effect appeared more evident when FA was loaded in Nanostructured Lipid Carriers (NLCs), used as a drug delivery system. A relationship between apoptotic pathway activation and changes of DL emission was speculated, too^35^.

Herein, we assessed the effects of FA, as free compound, or loaded in NLCs (FA-NLCs) on U-87 MG cells by studying some cellular pathways, DL emission and their correlation, in order to highlight their possible use for GBM therapy. Particular attention was focused on the effect of blank NLCs. Bcl-2, ERK1/2 and c-Myc expression levels were evaluated. Caspase-3 and PARP-1 cleavages were also tested to detect the apoptotic pathway activation.

## Results

### NLCs characterization and intracellular FA uptake into NLCs

Blank NLCs and FA-NLCs, having 150–200 nm of mean particles size of spherical shape (Supplementary Fig. S1a–c), were successfully prepared by the eco-friendly procedure exploited in this work among the different preparation procedures proposed in literature^34^. Both blank and FA-NLCs showed high values of phase inversion temperature (>75 °C), suggesting the good stability of the nanosuspensions^36^. Photon Correlation Spectroscopy (PCS) results showed the presence of homogeneous systems (polydispersity index, PDI < 0.3) with a slight decrease in mean particles size when loading the drug (Supplementary Fig. S1a). Interestingly, a slight decrease of zeta potential (ZP) value was observed when adding FA (Supplementary Fig. S1a), whose encapsulation efficiency (EE) value was found to be 90.5% (±0.94) and loading capacity (LC) was 38.97% (±0.53). A controlled drug release profile was observed (Supplementary Fig. S1d), thus confirming our previous results in which FA release from NLC was studied and compared to that of solid lipid nanoparticles and nanoemulsions^34^. Our previous studies performed on U-87 MG cells showed that NLCs incorporated into inner core the total amount of FA (1.2% w/w) without the occurrence of surface adsorption phenomena^37^.

### Cellular viability

To assess the effect of the treatment of U-87 MG cells with blank NLCs or FA or FA-NLCs on cellular viability, MTT bioassay was performed. The concentration of free FA, FA-NLCs and, therefore, blank NLCs, was performed in order to have final FA concentration 36 µM. The optimal exposure time of the cell line cultures was 24 h^34,37^. The results were expressed as the percentage of cellular viability. No significant differences between PBS and DMSO-treated U-87 MG cell cultures were found, then they were used as controls. The percentage of inhibition of cellular viability was compared with the controls taken as 100%. The treatment of the cells with blank NLCs did not induce a change in cellular viability, when compared with the controls. Free FA significantly decreased cellular viability (~70%), when compared with the controls. A strong reduction in cellular viability (~40%), when compared with the controls was evident after the FA-NLC exposure. These results confirm our previous observations^34,37^ and, for clearness, are reported in Supplementary Fig. S2.

### ERK1/2, c-Myc expression levels

**Figure 1:**
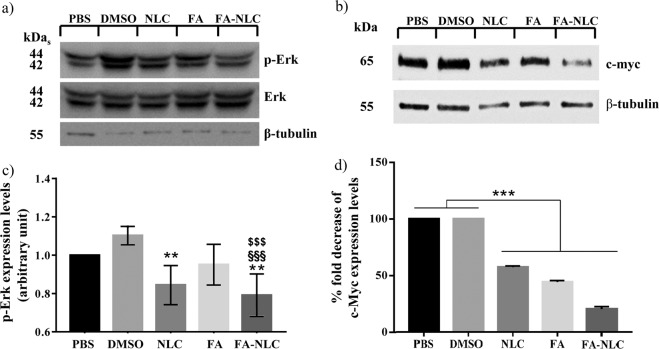
Synergic effect of FA and NLCs on ERK1/2, c-Myc expression levels. Representative immunoblots of (**a**) ERK and p-ERK and (**b**) c-Myc expression levels in U-87 MG human cell lines in the absence or in the presence of 36 µM of FA, blank NLCs or FA-NLCs, for 24 h. β-tubulin has been used to normalize protein expression levels. Cropped images of p-ERK, ERK and β-Tubulin have been obtained from the same filter after sequential exposure to the correspondent primary antibodies to analyse the changes in expression levels of phosphorylated protein versus the un-phosphorylated ones. Cropped images belonging to the same filter, obtained from other Western Blot, are also shown for c-Myc and β-Tubulin. Densitometric analysis of (**c)** phosphorylated ERK1/2 expression levels and (**d)** c-Myc expression levels in response to the treatments, when compared with untreated ones used as controls. The results (**c**,**d**) are reported as the mean ± S.D. of the values of four separated experiments performed in triplicate. Significant differences: **p < 0.01 DMSO vs NLC and FA-NLC; ***p < 0.001 samples vs controls; ^§§§^, ^$$$^p < 0.001 FA-NLC vs DMSO and vs FA, respectively.

Figure 1a,c show a representative immunoblot and densitometric analysis of ERK1/2 expression levels, performed in total cell lysates through Western blotting analyses of untreated and treated U-87 MG cells with blank NLCs or FA or FA-NLCs. Blank NLCs determined a reduction of p-ERK, when compared with the controls, while FA alone induced a decrease, barely significant (p < 0.33), of p-ERK. A high significant reduction was evident when cells were exposed to FA-NLCs. The expression levels of c-Myc were reported in Fig. 1b,d. The treatment of U-87 MG with blank NLCs, FA and FA-NLCs caused a highly significant reduction of c-Myc expression levels. The effect was particularly evident when the cells were exposed to FA-NLCs. Supplementary Figs. S3 and S4 report compliance with the digital image and integrity policies relative to Western Blotting analysis.

### Bcl-2 expression levels

**Figure 2:**
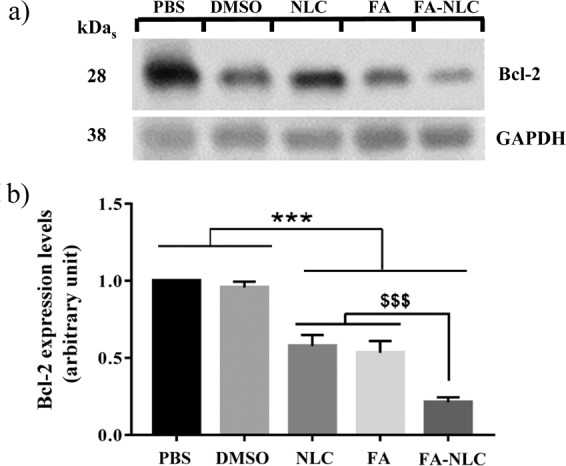
Synergic effect of FA and NLCs on Bcl-2 expression levels. (**a)** Representative cropped images of immunoblot of Bcl-2 expression levels after 24 h treatments on U-87 MG human cell lines with 36 µM of PBS, DMSO, blank NLCs, free FA or FA-NLCs. GAPDH has been used to normalize protein expression levels. Cropped images of Bcl-2 and GAPDH have been obtained from the same filter after sequential exposure to the correspondent primary antibodies to analyse possible changes in the expression levels of proteins. (**b**) Densitometric analysis of Bcl-2 in response to the treatments, when compared with the respective controls. Results are expressed as the mean ± S.D. of the values of four separated experiments performed in triplicate. ^***^p < 0.001 samples vs PBS and vs DMSO; ^$$$^p < 0.001 FA-NLC vs NLC and vs FA.

To investigate how treatment of U-87 MG cells with blank NLCs or free FA or FA-NLCs could affect the expression levels of Bcl-2, total cell lysates were analyzed through Western Blot analysis. No significant changes in the expression levels of Bcl-2 were observed in DMSO treated U-87 MG cells, when compared with PBS-treated ones. The treatments of U-87 MG cells with blank NLCs or FA induced a significant decrease in Bcl-2 expression levels when compared with their respective control. A stronger significant reduction of Bcl-2 expression levels was observed when cells were exposed to FA-NLCs (Fig. 2). Supplementary Fig. S5 reports compliance with the digital image and integrity policies relative to Western Blotting analysis.

### Apoptotic pathway assessment

To assess the effect of or blank NLCs or FA or FA-NLCs on the apoptotic pathway, caspase-3 and PARP-1 cleavage were evaluated by indirect immunofluorescence.

**Figure 3:**
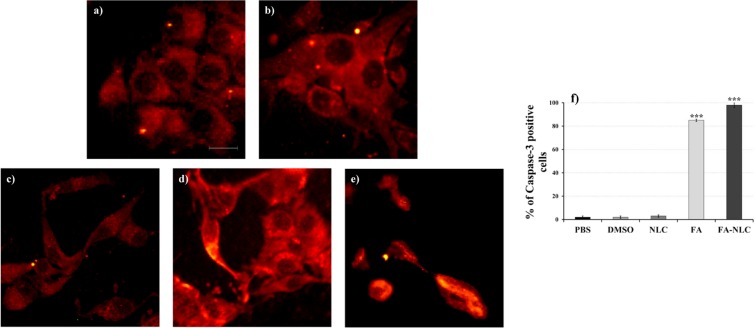
Effect of FA and FA-NLCs on Caspase-3 cleavage. Immunocytochemical analysis of caspase-3 cleavage, through fluorescent microscopy, in U-87 MG treated for 24 h with 36 µM of: (**a**) PBS, (**b**) DMSO, (**c**) blank NLCs, (**d**) free FA, (**e**) FA-NLCs. Scale bars = 50 µm. (**f**) Quantification and statistical analysis of caspase-3 cleveage. Results are expressed as the mean ± S.D. of the values collected from 4 fields/coverslips of the values of four separated experiments performed in triplicate. ***p < 0.001 FA and FA-NLC versus PBS, DMSO and NLC.

Figure 3a–e shows a representative picture of the caspase-3 cleavage performed in all the experimental conditions of exposed U-87 MG cells through immunocytochemical techniques and visualization to fluorescence microscopy. No significant number of positive cells for caspase-3 cleavage in cell cultures treated with DMSO and blank NLCs was observed (Fig. 3b,c), when compared with the PBS control (Fig. 3a). In contrast, a significant increase in the number of positive cells for caspase-3 cleavage in the cell cultures exposed to FA was found (Fig. 3d). The effect was more evident when the cells were exposed to FA-NLC in which all the cells showed apoptotic morphology (Fig. 3e). These observations were confirmed through quantification and statistical analysis of caspase-3 cleavage immunolabeling (Fig. 3f).

**Figure 4:**
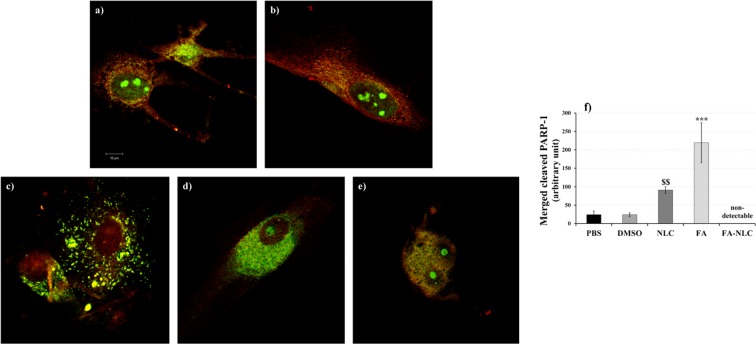
Synergic effect of FA and NLCs on PARP-1 localization and expression. Representative panel relative to PARP-1 expression and localization performed on single cell analyzed through confocal laser scanning microscopy of U-87 MG human cell lines treated for 24 h with 36 µM of: (**a**) PBS, (**b**) DMSO, (**c**) blank NLCs, (**d**) free FA, (**e**) FA-NLCs. Green or red colours show uncleaved PARP-1, yellow colour shows cleaved PARP-1. Scale bars = 10 µm. (**f**) Quantification and statistical analysis of merged cleaved PARP-1. Data are expressed as the mean ± S.E. of the values obtained from ten fields/coverslip of four separate experiments performed in triplicate. ***p < 0.0001 FA versus all conditions, ^$$^p < 0.005 NLCs versus PBS and DMSO.

Dual-channel Confocal Laser Scanning Microscopy (CLSM) analysis was used for imaging of double immunofluorescence for PARP-1 intracellular localization and expression on single cell (Fig. 4). Figure 4a–e shows a representative panel of confocal images of U-87 MG cells incubated with FITC-conjugated polyclonal antibody against PARP-1 (green) and TRITC-conjugated polyclonal antibody against PARP-1 (red): the merged images demonstrated the co-localization of cleaved-PARP-1 into the cells (yellow). In control cells PARP-1 appeared localized in the cytosol and the nuclear compartment. Furthermore, the nucleoli were positive for the protein (Fig. 4a,b).

In U-87 MG cells treated with blank NLCs, a positivity for cleaved PARP-1 (yellow) in the cytosol and mitochondria was found, which appeared significantly increased when compared with the controls. A low positivity of the cells for uncleaved protein (red) into the nuclear compartment was observed, when compared with the controls (Fig. 4c).

CLSM analysis performed on FA treated cells showed a strong positivity for cleaved PARP-1 (yellow) in the cytosol and a light signal in the nucleus. The effect was also accompanied by the change in cellular morphology, which had a roundish appearance (Fig. 4d), when compared with the control and blank NLCs treated ones. When the cells were exposed to FA-NLCs, a higher positivity for cleaved PARP-1 both in the cytosol and nuclear compartment was found and all the cells showed an apoptotic feature (Fig. 4e).

No non-specific staining of U-87 MG cells was observed in control incubations in which the primary antibody was omitted.

The quantification and statistical analysis of cleaved PARP-1 immunolabeling obtained and collected from ten fields/coverslip of four separate experiments performed in triplicate was reported in Fig. 4f. Blank NLC and free FA induced a significant increase of cleaved PARP-1. Since cells treated with FA-NLC were all in apoptosis, it was not possible to quantify cleaved PARP-1.

### Delayed luminescence results

**Figure 5:**
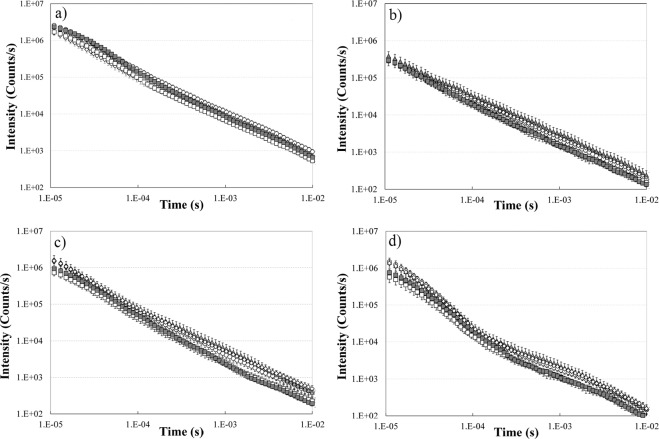
Time trends of Delayed Luminescence. Time trend of DL emitted by U-87 MG cell lines cultures in (**a**) visible range (350–850 nm), (**b**) blue region (425–475 nm), (**c**) green/yellow region (525–575 nm) and (**d**) red region (625–675 nm) treated with: (open circle) PBS; (diamond) DMSO; (open square) blank NLCs; (grey triangle) FA; (grey square) FA-NLCs. Results are expressed as the mean ± S.E. of the values of at least three biological replicates in triplicate.

The DL emitted from U-87 MG cells treated with PBS (used as control), DMSO, blank NLCs, free FA, FA-NLCs was recorded in the time interval 10 µs −10 ms after switching off the UVA laser excitation and evaluated in visible range (350 ÷ 850 nm) and three spectral regions: blue (425 ÷ 475 nm), green/yellow (525 ÷ 575 nm) and red (625 ÷ 675 nm). The corresponding DL time decays are reported in Fig. 5. It appears that (Fig. 5b–d) the presence of NLCs in the sample induced significant changes, with respect to other samples, in the time trends of the DL spectral components. These results cannot be ascribed to difference in cellular density^18^ due to the fact that in the whole visible range only slight differences are revealed both in DL time trends (Fig. 5a) and in the DL-integral for each DL decay kinetics, DLI, that is the total number of photons emitted (see Supplementary Fig. S6), from cell cultures after treatments with DMSO, FA and FA-NLCs when compared with the ones treated with PBS.

**Figure 6:**
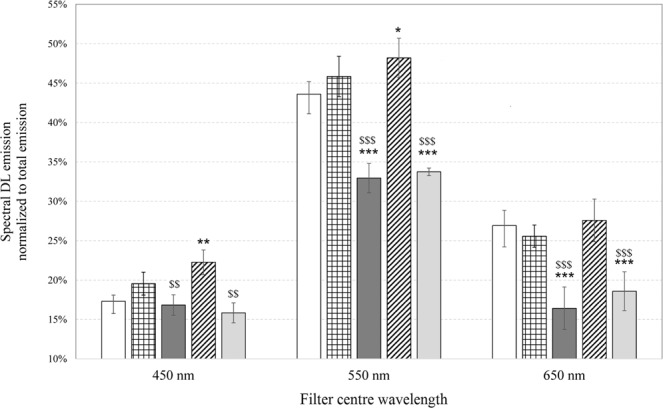
Normalized emission spectra of Delayed Luminescence. Spectral DL-integral emission (DLI_λ_) normalized to respective DL-integral emission in visible range (DLI_vis_) of U-87 MG cell cultures treated with: (white bar) PBS; (squared bar) DMSO; (dark grey bar) blank NLCs; (hatched bar) FA; (light grey bar) FA-NLCs. Results are expressed as the mean ± S.E. of the values of at least three biological replicates in triplicate. Significant differences: ^*^p < 0.05, ^**^p < 0.01, ^***^p < 0.001 vs PBS; ^$^p < 0.05, ^$$^p < 0.01, ^$$$^p < 0.001 vs FA.

We denoted DLI_vis_, DLI_450_, DLI_550_ and DLI_650_, the DL-integral for each DL decay kinetics in visible, blue, green/yellow and red regions respectively (spectral intervals were denoted with their central wavelength). To better evaluate the effect of treatments, each DLI_λ_ at a given central wavelength λ was normalized to DLI_vis_ (Fig. 6). Data reported in Fig. 6 show that the effects of NLCs and FA-NLCs on cell cultures are: (i) a highly significant reduction, when compared with the effects of PBS, DMSO and FA, on the emission ratios DLI_550_/DLI_vis_ and DLI_650_/DLI_vis,_ and (ii) a significant decrease, with respect the effect of FA, on DLI_450_/DLI_vis_. Moreover, the effect of FA treatment is a significant increase, with respect to PBS treated cell cultures, on DLI_450_/DLI_vis_ and DLI_550_/DLI_vis_. The analysis of Fig. 6 reveals that DL-integral spectral emission DLI_λ_ of U-87 MG cell cultures treated with NLCs unexpectedly shifted outside the spectral regions detectable with the used pass-band filters (centred at 450 nm, 550 nm and 650 nm) when compared with other samples. To stress this point, Table 1 shows the percentage ratio of the sum of DLI_450_, DLI_550_ and DLI_650_ to DLI_vis_ for each sample.

**Table 1:** Shift of Delayed Luminescence spectral emission as function of the treatments.

U-87 MG	(DLI_450_ + DLI_550_ + DLI_650_)/DLI_vis_
+PBS	87.8% ± 1.9
+DMSO	91.0% ± 2.6
+NLC	66.2% ± 2.7
+FA	98.1% ± 2.5
+FA-NLC	68.2% ± 2.5

Percentage ratio of the sum of DL-integral values obtained by using the pass band filters centred at 450 nm, 550 nm, 650 nm to the respective DL-integral acquired in the visible range (350–850 nm). Data are expressed as mean ± S.E. of the values of at least three biological replicates in triplicate.

**Figure 7:**
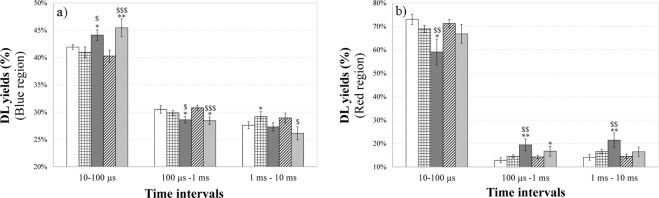
Normalized yields of spectral Delayed Luminescence emission as a function of the integration time intervals. DL yields in the (**a**) blue and (**b**) red spectral regions from cell cultures in different time intervals of the temporal decay: 10–100 µs; 100 µs-1ms; 1–10 ms. U-87 MG cell line cultures treated with: (white bar) PBS; (squared bar) DMSO; (dark grey bar) blank NLCs; (hatched bar) FA; (light grey bar) FA-NLCs. Results are expressed as mean ± S.E. of the values of at least three biological replicates in triplicate. Significant differences: ^*^p < 0.05, ^***^p < 0.001 samples vs PBS; ^$^p < 0.05, ^$$$^p < 0.001 samples vs FA.

According to Fig. 5, all the time trends show a multimodal behaviour depending on the treatment. Every trend can be modelled through the sum of a few Becquerel functions (compressed hyperbolas), appropriately weighted, which are commonly used to describe decays of complex systems^38^. To better analyse and compare different DL kinetics, it resulted useful, as reported in previous papers^22,23^, consider, inside each time decay, the DL-integrals corresponding to the time intervals 10–100 µs, 100 µs-1 ms and 1–10 ms, denoting them DL-I, DL-II and DL-III respectively. This analysis reflects the presence of different emitting species, characterized by different lifetimes, that could be related to different electron transfer steps in MRC Complex I^26^. More precisely each DL-integrals, related to a certain time interval of the decay, was normalized to the corresponding DLI value related to the whole time interval (10 µs −10 ms) of the decay, that is, in the case of emission in blue region, the ratios DL-I_450_/DLI_450,_ DL-II_450_/DLI_450,_ DL-III_450_/DLI_450_ were analysed. Figure 7 compares these DL yields, in the blue and red regions of the DL emission spectra respectively, in the case of U-87 MG cells treated with PBS (control), DMSO, blank NLCs, FA, FA-NLCs.

The results showed that DL yields related to U-87 MG cells treated with DMSO and FA were comparable to those of untreated ones. On the contrary, when the cells were exposed to NLCs, also loading FA, a slight increase of DL-I and a slight decrease of DL-II and DL-III yields in the blue region were observed. No significant difference in the green/yellow region, except for DL-III_550_/DLI_550_ of FA-NLC treated cells, were found (see Supplementary Fig. S7). Moreover, the treatment of cell cultures with NLCs induced a decrease in DL-I_650_/DLI_650_ yield and a more relevant increase of DL-II_650_/DLI_650_ and DL-III_650_/DLI_650_. This emission at longer time intervals was slightly quenched in presence of FA loaded in NLCs.

## Discussion

It has been demonstrated that, in GBM and other cancers, mitochondria play a pivotal role for their involvement in several cellular processes, including energetic metabolism and resistance to apoptosis. Mitochondria also represent the major site for the generation of Reactive Oxygen Species (ROS), as well as superoxide anion $({{O}_{2}}^{-.})$, hydroxyl radical (OH^•^) and singlet oxygen (^1^O_2_)^39,40^. In particular, ROS production is mainly carried out at the level of the Complex I (NADH dehydrogenase) and Complex III (ubiquinol-cytochrome c reductase) of the electron transport chain. Furthermore, GBM and other cancer cells possess high basal levels of ROS that are able to stimulate several intracellular signal events, including ERK1/2 pathway, several oncogenes, as well as c-Myc^41^, responsible of the aberrant cell proliferation^39,42^. In addition, it has been postulated that the alterations of redox balance, with an increase in ROS production, can induce apoptosis^43^. Other evidences reported that flavonoids are able to induce apoptosis in GBM by activating several pathways, down-regulating the expression level of Blc-2^44^, suggesting that the modulation of ROS levels could represent a new strategy for its therapy.

An abundant phenolic phytochemical present in vegetables and fruits is FA that represents a therapeutic antioxidant able to induce cell cycle arrest and autophagy^45^. The anticancer effect of FA is due to its ability to inhibit anti-apoptotic proteins, such as Bcl-2, PARP-1, PI3K/AKT and ERK1/2 pathways, reducing also the expression of the cell cycle-related proteins (CDK_2_, CDK_4_ and CDK_6_)^46^. However, in the treatment of GBM and other brain diseases, the presence of the BBB obstacles the entry of the drugs into the Central Nervous System (CNS). Therefore, we developed a formulation of NLC, which represents a good strategy for FA delivery due to the small particles size and high homogeneity, the high long-term stability, the good systemic tolerability, the high encapsulation efficiency and controlled drug release properties^34,47,48^. To obtain NLC for the glioblastoma treatment, glyceryl oleate was selected for its bioadhesive properties which together with the well-known role of brij components in increasing drug targeting to the CNS, could enhance nanoparticles crossing through the BBB and selectively destroy tumour cells^49,50^. As reported in different articles, brij-decorated nanoparticles have been demonstrated to increase drug targeting to CNS^51–54^. On the other side, cetyl palmitate (CP) was selected as solid lipid to exploit its ability in inducing apoptosis^55^, thus turning it to advantage of glioblastoma treatment when using CP as raw material in the production of FA-loaded NLCs.

This study is focused on the evaluation of the effect of blank NLCs, free FA and FA loaded NLCs in U-87 MG human glioblastoma cell line through molecular and bioptical techniques. To assess the mitochondria status, the expression levels of some proteins, as well as Bcl-2 and PARP-1, involved in the inhibition of apoptosis and in aberrant cell growth (ERK1/2, c-Myc) of GBM were evaluated. The effect of treatment of cell line cultures with blank NLCs was also tested. The molecular results were correlated with DL emission.

The de-regulation of apoptosis is involved in many types of cancers, including GBM, and it is tightly connected with Bcl-2 family proteins, which are localized in the mitochondrial membranes, and are able to block the release of the calcium stores in the cytosol, controlling also the release of cell death factors, as well as Apoptosis-Inducing Factor and endonuclease G. Furthermore, Bcl-2 family regulates mitochondrial ROS production, cancer cell invasion and metastasis^56^. Bcl-2 interacts with PARP1, blocking PARP1 enzymatic activity and suppressing PARP1-dependent repair. This interaction might represent a potential therapeutic approach for Bcl-2-expressing tumours resistant to apoptosis, including GBM^10^. PARP1 is related to the increase of the intracellular calcium levels and to the activation of ERK pathway, which in turn leads to increased c-Myc expression levels, resulting in the aberrant cellular proliferation in GBM. PARP-1 activation through direct interaction with ERK2 promotes growth cellular proliferation amplifying ERK signalling which has as target the core histone acetylation and the expression of early gene in cancers^57^.

Our data show that free FA induced down-regulation of Bcl-2 expression, accompanied by caspase- 3 and PARP-1 cleavage. The treatment of U-87 MG cells with blank NLCs did not exert cytotoxicity or activate caspase-3 cleavage^34^, even if, surprisingly, a significant decrease in Blc-2 expression levels was observed. Furthermore, a low decrease of the positivity of the cells for PARP-1 in the nucleus was found (Fig. 4c), and the cleaved protein appeared prevalently localized in the cytosol and in mitochondria, when compared with the control cells (Fig. 4a,b). In addition, the exposure of cell line cultures to blank NLCs and FA loaded in NLCs caused a significant decrease in Bcl-2 expression levels and cleavage of caspase-3 and PARP-1, which were accompanied by the decrease of p-ERK expression levels. In contrast, our results demonstrate, for the first time, that free FA did not induce significant change in p-ERK expression in U-87 MG cells. When it was encapsulated into NLCs, it was able to significantly reduce ERKs phosphorylation expression levels. We also found that free FA and blank NLC treatment caused a significant decrease in c-Myc expression levels, that plays important functions in cell proliferation and induction of apoptosis. The effect was more evident when FA was loaded in NLCs.

The small change found in ERK pathway after the exposure of the cell to free FA, may be due to the poor solubility of FA^58^, that is not able to modulate ERK phosphorylation, in our experimental conditions. This hypothesis is supported by data obtained by Wang *et al*.^46^ which demonstrated that FA on osteosarcoma cells inhibits cellular proliferation, induces apoptosis down-regulating Bcl-2 and c-Myc expression levels through PI3K/Akt pathway. So, the strong decrease on c-Myc expression level induced by treatment with free FA might be due to different signal pathways involved in the activation of the transcription factor^59^.

The effects of the treatments on U-87 MG cells were also analysed by DL emission studies. Till now the studies regarding DL from mammalian cells highlighted that mitochondria could represent the primary source of this emission^21–24^. In particular, a connection between DL characteristics and functionality of the MRC-Complex I has been suggested^26^. For this reason, DL spectral emissions have been evaluated in the blue, green/yellow and red regions where mitochondrial biomarkers, such as reduced nicotinamide adenine dinucleotide (NADH), flavin mononucleotide (FMN), protoporphyrin IX and singlet oxygen (^1^O_2_) dimols emit^60–62^. We found that the treatment of cell cultures with FA affected very slightly DL intensities and kinetics, both in visible and spectral regions. In contrast, the exposure of U-87 MG cells to blank NLCs induced a more significant change of DL time trend decays and a shift of the DL spectral emission range (see Table 1). The latter effect might be related to the possible interaction of NLCs with mitochondria^63^. Although NLCs did not induce cytotoxicity and caspase-3 cleavage, the effect on DL changes could be due to the cationic formulation of NLCs that interact with lipids, proteins, and other components of the cell membranes. More precisely, NCLs were able to cause a significant down-regulation of Bcl-2 expression levels and a, even if low, cleavage of PARP-1, which could reflect a mitochondrial impairment. So, DL data support the idea that cationic NLCs^64^ were able to sensitize glioblastoma cells to the treatment, yielding significant improvements over the untreated cells. On the other hand, the reduced effect of FA treatment on U-87 MG cells observed on DL emission supports the idea that FA could not enter into mitochondria, but could be able to activate apoptosis and block cell cycle progression by directly acting on DNA^65^. In fact, free FA induced apoptosis pathway activation, as revealed by the increase of caspase-3 and PARP-1 cleavage, reducing also the expression levels of the nuclear transcription factor c-Myc. We also speculate that changes in DL temporal kinetics and DL yields, observed in the blue spectral region, could be used to assess the effect of blank NLCs and FA-NLCs on NAD-dependent pathways, reflecting the effect of NLC lipid matrix on the MRC. In particular, the increase of DL-I_450_/DLI_450_ ratio and the decrease of DL-II_450_/DLI_450_ ratio in U-87 MG cells treated with blank NLCs and FA-NLCs could be due to a higher NADH luminescence intensity characterized by shorter lifetimes. This result suggests, in accordance with what is reported in ref. ^66^, that NLCs treatment induces a lower mitochondrial respiratory function. Furthermore an increase of the NADH/NAD^+^ ratio (fluorescent and not fluorescent form, respectively) could be speculated, owing to its ability to influence the activities of the PARP-1, which uses NAD^+^ as substrate^67^. This hypothesis is also in agreement with the suggestion^68^ of a positive loop between c-Myc, whose expression levels decrease in NCLs and FA-NCLs treated cells (see Fig. 1b,d) and nicotinamide phosphoribosyltransferase (NAMPT), the enzyme that limits NAD^+^ synthesis rate in mammalian cells. As it regards the changes of DL yields in the red region (Fig. 7b), the significant decrease of DL-I_650_/DLI_650_ and increase of DL-II_650_/DLI_650_ and DL-III_650_/DLI_650_, when cells are treated with blank NLCs, could be connected to both ROS, specifically singlet oxygen, and protoporphyrin IX (PpIX) emission. A PpIX accumulation has been observed in cancer cells following inhibition of mitogen-activated protein kinase^69^. So, the multimodal behaviour observed in DL time trends in the red region can be due to more classes of light-emitting states. The decrease of DL yield at shorter time (DL-I_650_/DLI_650_) and the increase at longer times (DL-II_650_/DLI_650_ and DL-III_650_/DLI_650_) could be ascribed to, respectively: i) the oxygen-dependent quenching of DL emitted from PpIX^24,25,70^; ii) the enhancement of the dimol photoemission generated by colliding molecules of singlet oxygen produced at Complex I of MRC^26^. On the contrary, DL-II_650_/DLI_650_ and DL-III_650_/DLI_650_ decrease in FA-NLCs U-87 MG treated cells could be explained looking to the scavenger activity towards free radicals of FA when it was carried into mitochondrion through NLCs.

Taken together molecular and bioptical data, we speculate that the exposure of U-87 MG cells to free FA could activate apoptosis through the PI3K/Akt pathway, that in turn deregulating c-Myc and Bcl-2 expression levels, could lead to PARP-1 cleavage and so DNA damage. Blank NLCs could affect mitochondria functionality by, probably modifying, due to their solid lipid formulation, the respiratory control that is shifted versus to increased NADH levels, so inducing also an aberrant ROS production. The alteration of redox balance, in favour of oxidant, even if is not able to induce caspase-3 cleavage, activates ERK pathway that, in turn, induces a decrease in c-Myc and Bcl-2 expression levels, and PARP-1 cleavage.

The treatment with FA loaded in NLCs was able to activate apoptosis, accompanied by PARP-1 cleavage, down-regulation of ERK pathway, c-Myc and Blc-2 expression levels. This finding can be explained observing that FA encapsulation into NLCs and subsequent release increases the availability of antioxidants in cells and, perhaps, in mitochondria. In the latter case, both NLCs and FA could be able to exert, respectively, their pro-oxidant and anti-oxidant effect, with an imbalance towards the first, as observed by DL. Therefore, our data suggest a synergic pro-apoptotic effect performed by FA, released into the cells, and NLCs, penetrated into mitochondria, on different signalling pathways that lead U-87 MG cells to apoptosis.

## Conclusion

Our finding demonstrated a novel and promising interaction between NLC formulation and FA for the treatments of U-87 MG human glioblastoma cells and the ability of the technique based on DL acquisition to discriminate the effects induced by treatments on mitochondria functionality. In our experimental conditions, free FA induced a significant decrease in c-Myc and Bcl-2 expression levels accompanied by the activation of the apoptotic pathway. Blank NLCs, despite not inducing cytotoxicity, were able to make glioma cell cultures more sensitive to apoptosis, exercising a down-regulation of Blc-2, suppressing PARP-1 dependent repair and blocking cellular proliferation. The changes in DL emission suggest that NLCs affect mitochondria respiration, by inducing also an increase in ROS levels. Furthermore, we demonstrated that FA encapsulation into NLCs and following release may improve the anticancer activity in U-87 MG human glioblastoma cells, and DL emission correlation to molecular results highlights the synergic pro-apoptotic effect between FA and NLCs. Taken together our data clearly revealed that when FA is loaded in NLCs it was able to induce apoptosis and reduce ERK pathway and c-Myc expression, playing an important function in cell proliferation and apoptosis induction.

Thus, FA encapsulation into NLCs represents a promising tool for glioblastoma treatment according the pro-apoptotic effects observed in this *in vitro* study. Worth to note that the effective targeting properties and BBB crossing abilities could be assessed by researches performed or *in vitro* model of the BBB or *in vivo*. Studies are now in progress to better understand the change in mitochondrial status induced by NLCs both in absence and in presence of Ferulic Acid. In addition, other research activities will be pulsed to confirm the suitability of NLCs in enhancing FA overcoming of BBB.

## Materials and Methods

### Materials

Cetyl Palmitate (Cutina CP) was from BASF Italia S.p.A. (Cesano Maderno, MB, Italy). Oleth-20 (Brij 98), isopropyl stearate (IPS) and Gliceryl Oleate (Tegin O), were from A.C.E.F. S.p.a. (Piacenza, Italy). Ferulic Acid and Ceteth-20 (Brij 58) were provided by Fluka (Milan, Italy). Didodecyldimethylammonium bromide (DDAB), leupeptin, aprotinin, phenylmethylsulfonyl fluoride (PMSF), EDTA, EGTA, Sodium Dodecyl Sulfate (SDS) and phosphatase inhibitor cocktail II were from Sigma-Aldrich (Milan, Italy). Sodium Pyruvate, Isoceteth-20 (Arlasolve 200), 3(4,5-dimethyl-thiazol-2-yl)2,5-diphenyl-tetrazolium bromide (MTT), Dimethyl sulfoxide (DMSO), Lab-Tek II Chamber-Slide Systems, tetrarhodamine Isothiocyanfluorescein Isothiocyanate (FITC)-conjugated anti-mouse IgG polyclonal antibody and others analytical chemicals were purchased from Sigma–Aldrich (Milan, Italy). Regenerated cellulose membranes (Spectra/Por CE; Mol. Wet. Cut off 3000) were brought by Spectrum (Los Angeles, CA). Water, acetic acid and methanol were of LC grade and purchased from Merck (Milan, Italy). All other reagents were of analytical grade. U-87 MG human glioblastoma cell line was from Cell Bank Interlab Cell Line Collection (Genova, Italy). Trypsin, antibiotics, non-Essential Amino Acids, health inactivated Fetal Bovine Serum (GIBCO), Phosphate Buffer Saline solution (PBS), Normal Goat Serum (NGS, GIBCO), Modified Eagle Medium (MEM) with 2 mM GlutaMAX (GIBCO), WesternBreeze Chemiluminescent Western Blot Immunodetection Kit were from Invitrogen (Milan, Italy). Bicinchoninic acid Protein Assay Kit was from Pierce (Thermo Fisher Scientific, Milan, Italy). Rabbit monoclonal against Bcl-2, rabbit monoclonal against ERK1/2 (unphosphorylated), rabbit monoclonal against phosphorylated-ERK1/2, rabbit monoclonal antibody against β-Tubulin and rabbit polyclonal against Glyceraldehyde-3-phosphate dehydrogenase (GAPDH) were from Cell Signaling Technology (EuroClone, Milan, Italy). Mouse monoclonal against c-Myc was purchased by eBioscience (Prodotti Gianni, Milan, Italy). Mouse monoclonal against β-actin was from Santa Cruz Biotecnology Inc (Santa Cruz, CA, USA). Mouse monoclonal antibody against PARP was from Trevigen (Tema Ricerca s.r.l., Castenaso, Italy).

### NLC, preparation procedure, physical-chemical and technological characterization

NLC were prepared by Phase Inversion Temperature (PIT) method, a convenient eco-friendly procedure using low energy in heating^34^. CP/IPS (5% w/w) and 13% w/w of Brij 98 and Tegin O (2:1 ratio) were used as lipid and surfactant mixture, respectively. DDAB (0.5% w/w) was added to the lipid phase^71^. FA-loaded NLC were prepared adding the drug (0.7% w/w) to the oil phase. The conductivity meter Crison, mod. 525 (Modena, Italy), was used to detect the PIT temperature^72^. The mean particle size, PDI and ZP values of NLCs were determined after sample dilution (1:200) with ultra-purified water using the Zetasizer Nano S90 (Malvern Instruments, Malvern, UK)^72^. The amount of the encapsulated drug (EE%) and the drug loading capacity (LC%)were determined by ultracentrifugation for 1 hour at 1000 rpm (Beckman model J2–21 Centrifuge): the pellet was diluted in methanol, vortexed and filtrated (0.22 µm). Drug concentration was measured by HPLC. EE% and LC% were calculated as reported by ref. ^73^ using an HPLC Varian Prostar 230 (Varian, Milan, Italy) and a reversed-phase C18 column (Symmetry, 4.6 cm ×15 cm; Waters, Milan, Italy). The mobile phase consisted of methanol/CH_3_COOH (5% v/v) (60:40 v/v). FA calibration curve was constructed in the range 0.1–100 µg/ml (R^2^ = 0.9997). The instrument revealed any interference due to other components present in the NLCs. *In vitro* release experiments were performed using Franz-type diffusion cells^34^. Cryogenic transmission electron microscopy (Cryo-TEM) was used to characterize FA-NLC, using the procedure described in our previous study^37^.

### Glioblastoma cell line cultures

U-87 MG cell line was suspended and cultured in MEM containing 10% FBS, 1% Non-Essential Amino Acids, penicillin (50 U/mL), streptomycin (50 µg/mL), 2 mM Gluta-MAX, and 1 mM Sodium Pyruvate, plated in 75 cm^2^ flasks at a final density of 2 × 10^6^ cells and incubated at 37 °C in humidified atmosphere containing 5% CO_2_^35^. Every 2 or 3 days the medium was replaced. When the cultures were about 85–90% confluent, cells were subcultivated at 1:4 density ratio and incubated at 37 °C in humidified atmosphere containing 5% CO_2_.

### Treatment of glioblastoma cell line cultures

U-87 MG cell line cultures were exposed for 24 h to the different treatments: PBS, DMSO, free FA, blank NLCs and FA-loaded NLCs. Stock solution of free FA (7 mg/mL) diluted in DMSO, blank or FA-loaded NLCs (50 mg/ml referred to the solid lipid, FA concentration 0.7% w/w) diluted in PBS were prepared. For every test, the suitable aliquot from each stock solution was added to culture medium in order to obtain FA final concentration 36 µM, corresponding to a final NLC concentration 0.5 mg/mL. A group of cells was treated with a corresponding volume of PBS (final concentration 2% v/v) and used as control. Another group of cell cultures was treated with the corresponding volume of DMSO used to solubilize FA, having a final DMSO concentration of 2% v/v.

### MTT bioassay

Cell viability was monitored using MTT bioassay^74–76^. Results were expressed as a percentage of the control (PBS), taken as 100%, to normalize the different obtained values.

### Immunocytochemical assay

A group of U-87 MG cell lines were placed in Lab-Tek II Chamber-Slide Systems at the final density of 0.5 × 10^5^ cell/ml and incubated at 37 °C in a humidified 5% CO_2_–95% air mixture. About 80% confluent cell line cultures, were treated with PBS, DMSO, blank NLCs, free FA, FA-loaded NLCs and incubated at 37 °C in a humidified 5% CO_2_–95% air mixture for 24 h. After the treatment, the cell cultures were fixed for 20 min with 4% paraformaldehyde, washed for three times with PBS and incubated with 1% NGS for 1 h at 37 °C in humidified air and 5% CO_2_, to block unspecific sites^75^. Successively, they were incubated overnight, at 37 °C in humidified air and 5% CO_2_, with mouse monoclonal antibody against caspase-3 (diluted in PBS 1:100) or against PARP-1 (diluted in PBS 1:100). Cell cultures were then washed three times with PBS and incubated for 2 h with TRITC-conjugated anti-mouse IgG polyclonal antibody (1:64 in PBS) or FITC-conjugated anti-mouse IgG polyclonal antibody (1:64 in PBS). Finally, the cell cultures were washed three times with PBS, and the Lab-Tek II Chamber-Slide Systems were mounted in PBS/glycerol (50:50), and examined to visualize caspase-3 positive cells through Leica fluorescent microscope (Germany) and to analyze PARP-1 positive cells through Confocal Laser Scanning Microscope (CLSM, LSM-510 Meta, Zeiss, Germany). For the acquisition with CLSM, we used an Apo 63×/1.4 oil immersion objective and the Argon (λ = 488 nm) and HeNe (λ = 543 nm) lasers. Images were acquired at the pixel resolution of 1024×1024 and were processed to enhance brightness and contrast using the software Zen 2009. The version number for software ZEN 2009 was 5.5.0.452 and provided together ZEISS confocal microscope. The ZEN 2009 soft version is available at link https://www.softpedia.com/get/Multimedia/Graphic/Graphic-Viewers/ZEN-2009-Light-Edition.shtml. The optical fields were examined through simultaneous green and red fluorophore excitation. No non-specific staining of U-87 MG cancer cell lines was observed in control incubations in which the primary antibody was omitted^74–76^. The analysis of colocalization analysis of merged (yellow) cleaved PARP-1 (yellow) was made through with ImageJ software (version 2.0.0-rc-69/1.52p, free software downloaded from https://imagej.nih.gov/ij/download.html) by using the function Image Calculator, that, starting from two images and through the logical operation “AND”, return only the pixels appearing in the same position in all images.

### Western blotting analysis

Untreated and treated cells were lysed and protein concentration determined as in ref. ^77^. 50 µg of total proteins were separated through 8% or 12% SDS-PAGE. Filters obtained were then incubated with the following 1:1000 diluted primary antibodies: rabbit monoclonal against Bcl2, rabbit monoclonal against unphosphorylated ERK1/2, rabbit monoclonal against phosphorylated-ERK1/2, mouse monoclonal against c-Myc, rabbit monoclonal against β-tubulin and rabbit polyclonal against GAPDH. Anti-rabbit and anti-mouse secondary antibodies linked to alkaline phosphatase were then used. Immunoreactivity was detected using the WesternBreeze Chemiluminescent Western Blot Immunodetection Kit. Autoradiographic signals were captured through the VersaDoc Imaging System (Bio-Rad Laboratories Srl, Italy) and evaluated by densitometric analysis through the software Quantity One. The version number for software Quantity One was 4.6.7 and included with the Versadoc Instrument from BioRad Laboratories (https://www.bio-rad.com/it-it/product/quantity-one-1-d-analysis-software?ID=1de9eb3a-1eb5-4edb-82d2-68b91bf360fb).

### Delayed luminescence spectroscopy

The evaluation of DL from cell cultures has been performed using a suitable equipment with a single photon sensibility^35^. Briefly, each sample was stimulated using a laser pulse (Laser Photonics LN 230 C) at wavelength λ_exc_ = 337 nm, pulse-width 0.6 ns and energy13 ± 0.2 µJ/pulse. The DL optical photons (350–850 nm), were detected by a photomultiplier tube (Hamamatsu R7206-01 SEL) working, at temperature −10 °C, in single-photon counting mode and recorded using a Multi-Channel Scaler (ORTEC, Ametek, U.S.) as a function of arrival time (dwell time 2 µs). An electronic shutter was used to impose 10 µs time lag in acquisition after the laser pulse. The same run was repeated 100 times to enhance the difference in DL emissions. Spectral measurements were performed using Broadband Bandpass Interference Filter (Edmund Optics) centred at 450 nm, 550 nm and 650 nm (50 nm FWHM).

Before DL measure, immediately after the end of treatments, the medium was removed, the cells were detached by using 0.05% trypsin and 0.53 mM EDTA solution and incubated for 7 min at 37 °C. Trypsinization was stopped by adding 50% FBS, and the cells were centrifuged at 200 × g for 10 min. The obtained pellets were suspended in PBS, centrifuged (200 × g for 10 min) and suspended again in PBS at the final cell density ≥10^6^ cell/ml. DL spectroscopy was performed on single drops of 20 µl of such cell culture suspension at room temperature (21 ± 1 °C)

### Statistical data analysis

All analysis, including DL spectroscopy, were performed in triplicate or quadruplicate for each experimental condition and three or four-independent experiments were conducted. Data were statistically analysed using One-Way analysis of variance (ANOVA) followed by a post hoc Holm–Sidak test or by Tukey’s multiple comparisons test to estimate significant differences among groups. Data were expressed as mean ± S.D or, in the case of DL experiments and quantification analysis, as mean ± S.E. Statistical significance is reported in figure captions.

## Supplementary Information

Supplementary Information.

## Data Availability

All data supporting the findings on this study are included in this published article (and its Supplementary Information files). The datasets generated during and/or analysed during the current study are available from the corresponding autor on request.
