# Supplementary material for: Synergic pro-apoptotic effects of Ferulic Acid and nanostructured lipid carrier in glioblastoma cells assessed through molecular and Delayed Luminescence studies
Source: Sci Rep. 2020 Mar 13;10:4680. doi: 10.1038/s41598-020-61670-3 (PMC7070080; doi:10.1038/s41598-020-61670-3)
Supplement: Supplementary file 1 — Supplementary Information. [file 41598_2020_61670_MOESM1_ESM.pdf]

# Supplementary Information

## Synergic pro-apoptotic effects of Ferulic Acid and nanostructured lipid carrier in glioblastoma cells assessed through molecular and Delayed Luminescence studies

Rosaria Grasso[1,2,*], Paola Dell'Albani[3], Claudia Carbone[4], Michela Spatuzza[5], Roberta Bonfanti[3], Giovanni Sposito[6], Giovanni Puglisi[4], Francesco Musumeci[1,2], Agata Scordino[1,2], Agata Campisi[6,*]

[1]Department of Physics and Astronomy "Ettore Majorana", University of Catania, 95123, Catania, Italy

[2]Istituto Nazionale di Fisica Nucleare, Laboratori Nazionali del Sud, 95123, Catania, Italy

[3]Institute for Biomedical Research and Innovation, Italian National Research Council, 95126, Catania, Italy

[4]Department of Drug Sciences, Laboratory of Drug Delivery Technology, University of Catania, 95123, Catania, Italy

[5]Oasi Institute for Research on Mental Retardation and Brain Aging (IRCCS), 94018, Troina, Italy

[6]Department of Drug Sciences, Section of Biochemistry, University of Catania, 95123, Catania, Italy

*Corresponding to: rosaria.grasso@ct.infn.it; campisag@unict.it.

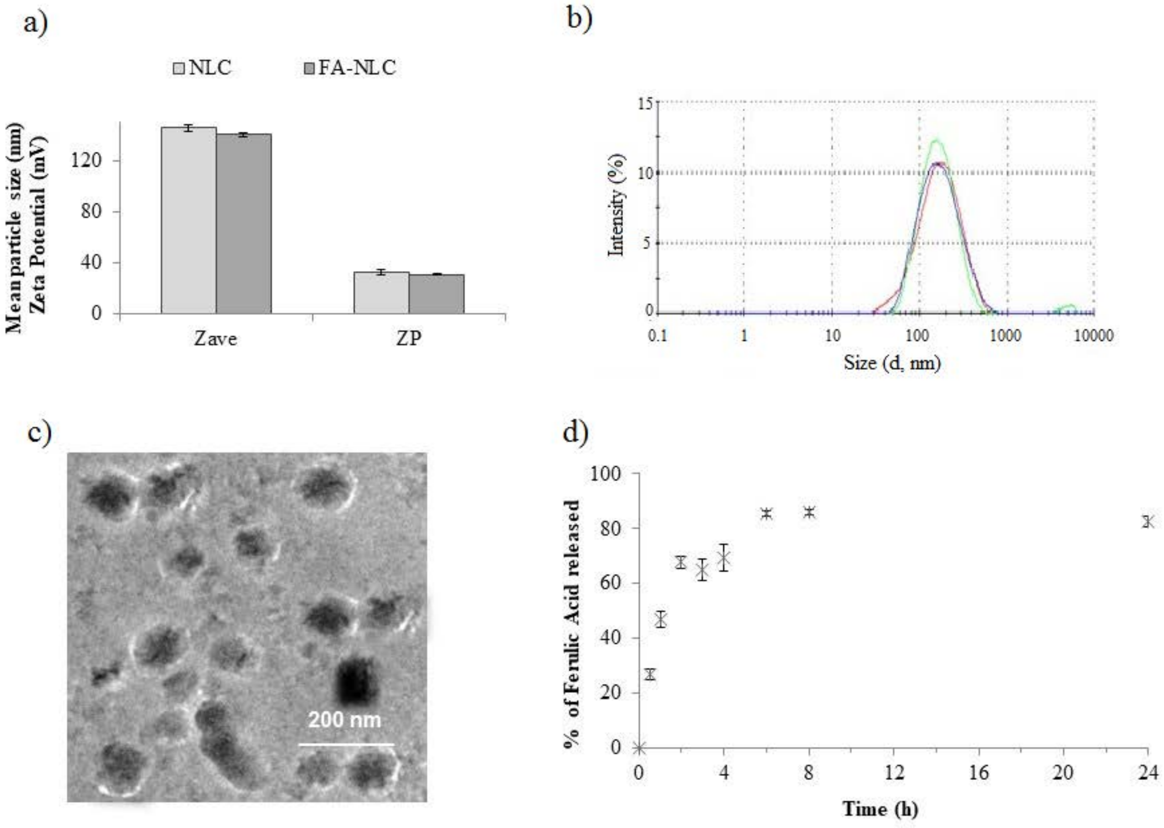


**Figure S1**: Physical-chemical characterization of FA-loaded NLC. a) Mean particle size (Zave) and Zeta potential (ZP) compared to unloaded NLC; b) size distribution of particles reported by Intensity obtained from three separated experiments; c) representative cryogenic transmission electron microscopy (Cryo-TEM) image of FA-NLC; d) percentage of FA released after 24 h. The data are reported as the mean ± S.D. of the values of three separated experiments performed in triplicate.

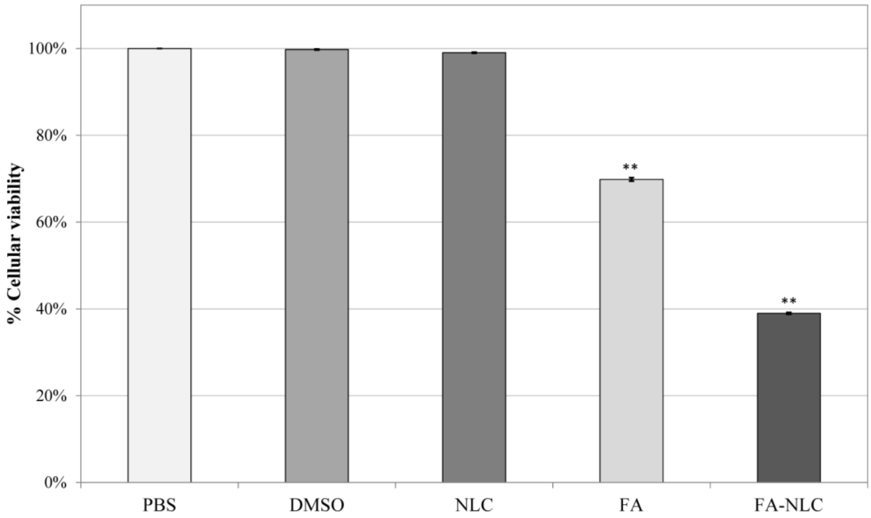


**Figure S2.** Percentage of cell viability performed through MTT assay in U-87 MG cell line cultures treated 24 h with 36 µM of DMSO, blank NLCs, FA and FA-NLCs. Results are expressed as mean ± S.D. of the values of four indipendent experiments performed in triplicate. $^{**}p<0.01$ significant vs controls.

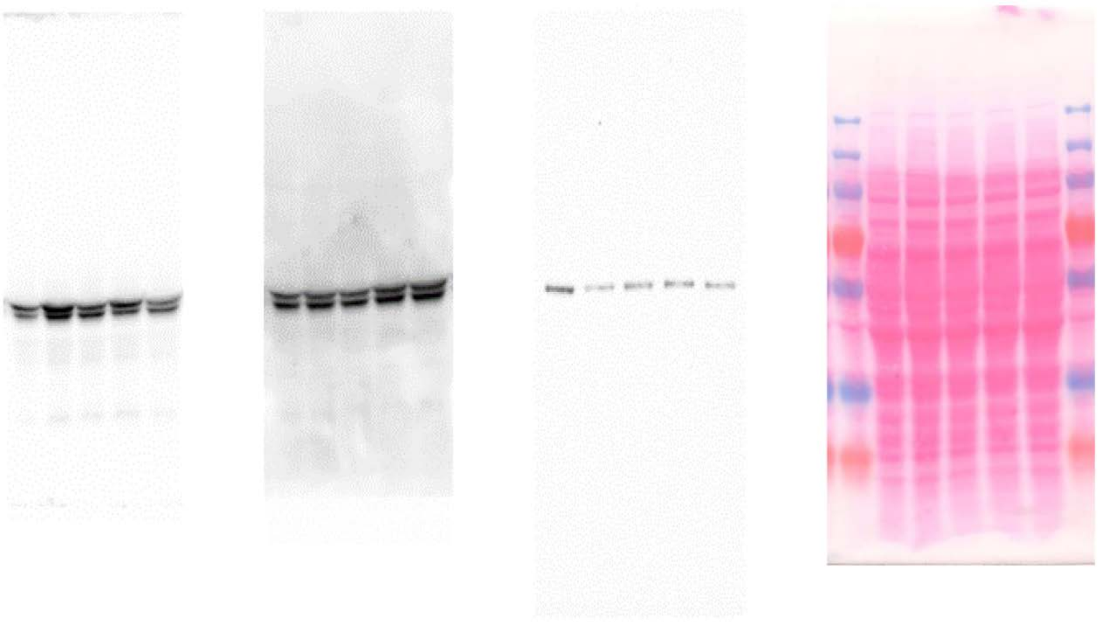


**Figure S3.** Synergic effect of FA and NLCs on ERK1/2 expression levels. Representative immunoblots of ERK and p-ERK expression levels in U-87 MG human cell lines in the absence or in the presence of 36 μM of FA, blank NLCs or FA-NLCs, for 24 h. The filter has been analysed first for p-ERK expression levels, than stripped re-bloked and probed for ERK expression levels. A further step was to check for the housekeeping gene β-Tubulin, then the filter was again stripped, blocked and probed for β-Tubulin. Primary antibodies were 1:1000 diluted. To analyse the specific protein expression levels the WesternBreeze Chemiluminescent Western Blot Immunodetection Kit was used.

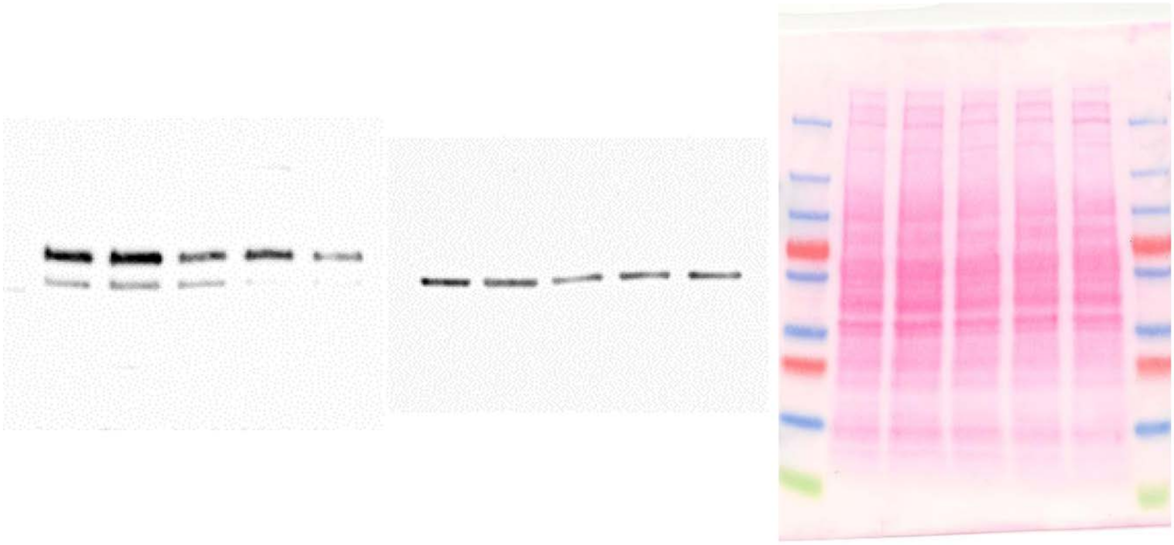

c-Myc β-Tubulin 4-20% SDS-PAGE
Red ponceau

**Figure S4.** Synergic effect of FA and NLCs on c-Myc expression levels. Representative immunoblots of c-Myc expression levels in U-87 MG human cell lines in the absence or in the presence of 36 µM of FA, blank NLCs or FA-NLCs, for 24 h. The filter has been analysed for c-Myc expression levels then probed for the housekeeping gene β-Tubulin. It was not necessary to strip-off the first antibody used to probe it for β-Tubulin, since the first Antibody was a mouse-anti-c-Myc, while the second one was rabbit-anti- β-Tubulin. Primary antibodies were 1:1000 diluted. To analyse the specific protein expression levels the WesternBreeze Chemiluminescent Western Blot Immunodetection Kit was used

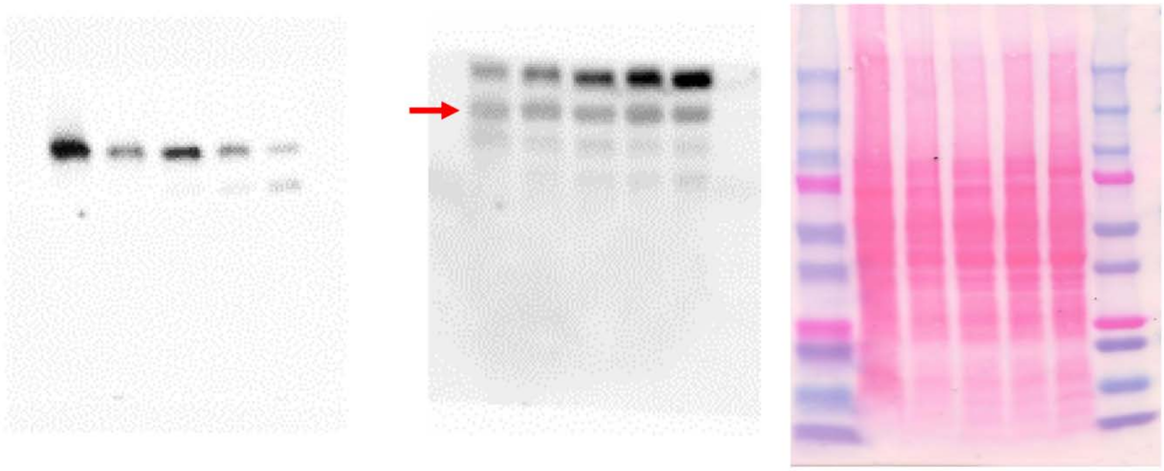

Bcl-2 GAPDH 12% SDS-PAGE
Red ponceau

**Figure S5.** Synergic effect of FA and NLCs on Bcl-2 expression levels. Representative cropped images of immunoblot of Bcl-2 expression levels after 24 h treatments U-87 MG human cell lines with 36 μM of PBS, DMSO, blank NLCs, free FA or FA-NLCs. GAPDH has been used to normalize protein expression levels. The filter has been analysed for Bcl-2 expression levels then probed for the housekeeping gene GAPDH. It was not necessary to strip-off the first antibody used to probe it for GAPDH, since the first Antibody was a rabbit polyclonal against GAPDH. Primary antibodies were 1:1000 diluted. To analyse the specific protein expression levels the WesternBreeze Chemiluminescent Western Blot Immunodetection Kit was used.

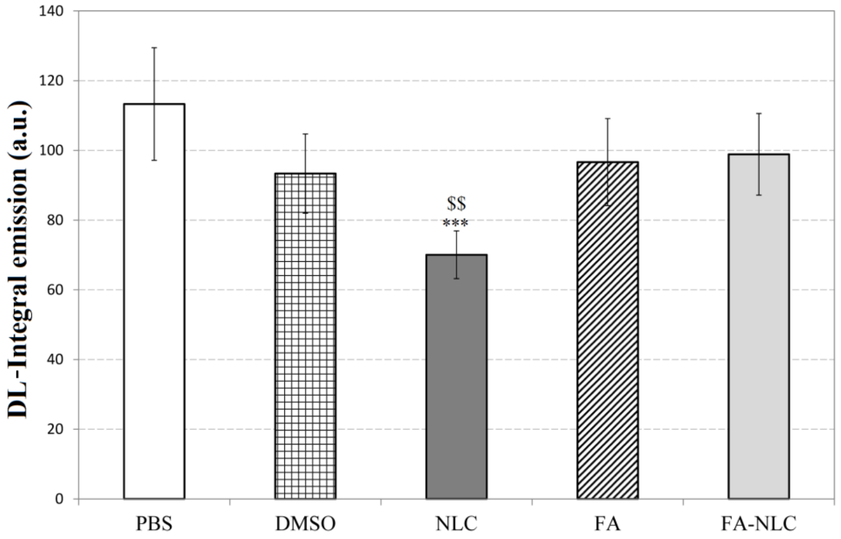


**Figure S6.** DL-integral emission in visible range, $DLI_{vis}$, of U-87 MG cell cultures treated with: (white bar) PBS; (squared bar) DMSO; (dark grey bar) blank NLCs; (hatched bar) FA; (light grey bar) FA-NLCs. Results are expressed as the mean ± S.E. of the values of at least three biological replicates in triplicate. Significant differences: $^{***}$p<0.001vs PBS; $^{\$\$}$p<0.01 vs FA.

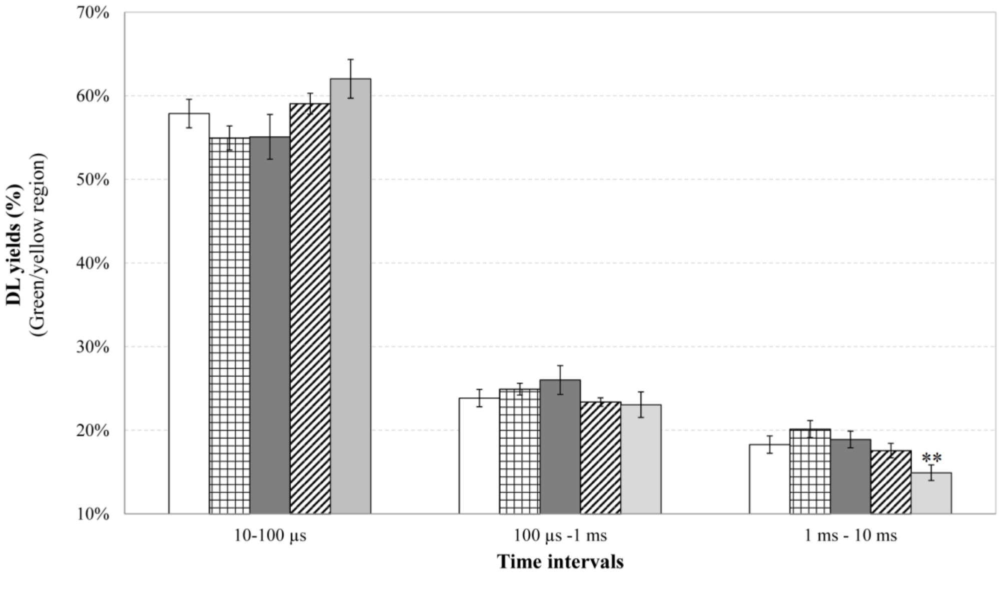


**Figure S7.** DL yields in the green/yellow region (525÷575 nm) from cell cultures in different integration time intervals of the temporal decay: 10-100µs; 100µs-1ms; 1-10ms. U-87 MG cell line cultures treated with: (white bar) PBS; (squared bar) DMSO; (dark grey bar) blank NLCs; (hatched bar) FA; (light grey bar) FA-NLCs. Results are expressed as mean ± S.E. of the values of at least three biological replicates in triplicate. Significant differences: $^{**}$p<0.01 samples vs PBS.
